# Current practice, barriers and facilitators to caries management in children: a qualitative study in the Netherlands

**DOI:** 10.1007/s40368-026-01202-1

**Published:** 2026-04-03

**Authors:** Catharina P. M. Elsenberg, Monique H. van der Veen, Sten Jeuring, Marie Charlotte D.N.J.M. Huysmans, Linda Kwakkenbos

**Affiliations:** 1 Department of Dentistry, Radboud University Nijmegen Medical Centre, Nijmegen, Netherlands; 2 Department of Oral Hygiene, Inholland University of Applied Sciences, Amsterdam, Netherlands; 3 Research Group Collaborating on Oral Health, Inholland University of Applied Sciences, Amsterdam, Netherlands; 4 Department of Paediatric Dentistry, Academic Center for Dentistry Amsterdam, Amsterdam, Netherlands; 5 Department of Clinical Psychology, Behavioural Science Institute, Radboud University Nijmegen, Nijmegen, Netherlands; 6 Department of Psychiatry, Radboud University Nijmegen, Nijmegen, Netherlands

**Keywords:** Children, Caries management, Barriers, Facilitators, Guideline

## Abstract

**Background:**

Dental caries remains a global challenge and implementing preventive and minimally invasive treatments for primary teeth is difficult in everyday practice. Evidence on how these approaches are applied and perceived is limited.

**Aim:**

Objectives were to (1) describe the current practice in preventive and minimally invasive caries treatment for children; (2) evaluate their alignment with national guidelines; and (3) identify barriers and facilitators experienced by oral healthcare professionals (OHCPs) and parents in managing caries in Dutch children under 12 years.

**Design:**

Semi-structured interviews were conducted with purposively sampled prevention assistants, dental hygienists, dentists, and parents. Data were analysed thematically using inductive and deductive approaches.

**Results:**

Twenty-six OHCPs and ten parents participated. OHCPs emphasised primary prevention through brushing and healthy eating. Care focussed on caries stabilisation and treatment, partly in accordance with the national guidelines, though advanced lesions were often treated restoratively. Behavioural modification strategies were used, but mostly without structure or training, mainly relying on information provision. OHCPs struggled to share responsibility with parents, though parents viewed themselves as accountable. Reported barriers included reimbursement systems, time constraints, parental influence, and limited familiarity with guideline-recommended options. Facilitators included cooperation, material availability, and continuing education.

**Conclusion:**

OCHPs aim to deliver preventive and minimally invasive caries management, but alignment with the national guideline is limited.

## Introduction

Dental caries remains prevalent and is considered the most preventable health problem worldwide (Shoaee et al. 2017). Within Europe, caries remains a common public health challenge (Benza et al. 2021). In the Netherlands, approximately 25% of 5 years old and 40% of 11 years old have at least one cavity exposing the dentine (Schuller et al. 2018). Comparable to other European countries, dental care in the Netherlands follows a uniform fee-for-service system (Ndayishimiye et al. 2025). Care for children up to 17 years is largely covered by the national health insurance. Nonetheless, limited attention has been paid to the primary dentition. Children often visit the dental practice at a relatively late age. Preventive care is frequently provided, but often not tailored to individual caries risk (Van Rijkom et al. 2004; Den Boer 2022). Due to a perceived overuse of preventive services in paediatric dentistry, the overall cost of care has increased, and restrictions have been introduced by insurance companies in recent years (KNMT 2024). If oral healthcare professionals (OHCPs) wish to claim beyond these limits (e.g., > 8 sealants/year > 1 h of preventive treatments in children) prior authorization is required.

Healthcare systems are commonly structured into primary and secondary care. In the Netherlands, paediatric dental referral clinics constitute secondary care for children referred from general dental practice. Primary care is delivered in private practices, ranging from solo dentists to group practices and, increasingly, dental chains, of which some focus exclusively on children (youth dental care organisations). Professionals providing oral care to children range from dentists (in referral practices often paedodontists), dental hygienists, who are trained for 4 years including diagnostic and restorative skills, and prevention assistants, with variable training (Den Boer 2022: KNMT 2022: NVM 2019). Although dental hygienists are usually employed within dental practices, they have a partially autonomous scope of practice. A 2020 pilot provisionally granted (registered) dental hygienists independent authority to indicate and perform specific procedures, including treatment of caries lesions and taking radiographs. Furthermore, within the primary dentition, they are permitted to place restorations and preformed metal crowns. Prevention assistants are authorised to independently perform tasks, such as providing oral health education, dietary advice, and oral hygiene instructions, as well as dental cleaning and applying fluoride (NVM 2019.

For a long time, prevention and non-operative management of dental caries has been guided by the Ivoren Kruis Advice on Prevention of Caries (Ivoren kruis 2021). Basic advice applies to all ages and includes twice-daily 2-min brushing with fluoride toothpaste from the eruption of the first tooth, parental assistance until age 12, and limiting sugar. Dietary guidance recommends up to five eating/drinking moments daily for children under five and seven for older children (Ivoren Kruis 2021).

Clinical guidelines are commonly developed to improve care. In the Netherlands, the clinical practice guidelines for children’s oral care include, ‘Diagnostic’ (2019), and ‘Prevention and treatment of caries’ (2020) (Kennisinstituut 2019; Kennisinstituut 2020). These guidelines align with international standards and promote a person-centred approach combined with minimally invasive caries management (Kennisinstituut 2019; Kennisinstituut 2020; Scottish Dental Clinical Effectiveness Programme 2025). The diagnostic guideline recommends using radiographs, alongside visual detection, when there is a suspected increased caries risk, with recall intervals tailored based on individual risk (Kennisinstituut 2019). The prevention and treatment guideline recommends individualised minimally invasive treatment strategies that minimise the burden on the child whilst optimising tooth prognosis (Kennisinstituut 2020).

Several Dutch preventive programmes for children, such as Johny Joker and Gewoon Gaaf (Simply Sound, based on the Nexø method) (Vermaire et al. 2014), promote risk-based caries management and minimal intervention dentistry using communication aids (Vermaire et al. 2014). Many international guidelines, including the Dutch guideline, recommend that OHCPs should identify unhealthy behaviours and motivate children and parents to modify unhealthy risk behaviours through motivational interviewing for behavioural change, due to its evidence-based efficacy in promoting healthy oral behaviours (Kennisinstituut 2020).

Extensive research has been conducted worldwide on the implementation of guidelines in dentistry. A study by Gruß et al. (2021) demonstrated that the implementation of clinical guidelines progresses slowly in the United Kingdom. Potential explanations include a lack of collaboration between researchers and OHCPs, as well as challenges in translating scientific knowledge into clinical practice. Findings from a study conducted by the World Dental Federation European Regional Organization (Yamalik et al. 2015) suggest that evidence-based practice is a relatively recent development in dentistry and remains only partially implemented. This study highlighted a clear need for additional resources to facilitate the integration of clinical guidelines into routine dental practice (Yamalik et al. 2015).

Implementation science demonstrates that even evidence-based interventions are not automatically adopted in clinical practice (Brouwers et al. 2011; Rapport et al. 2022). Successful guideline implementation requires the engagement of OHCPs (Guncu et al. 2018) and benefits further from patient involvement (Hosie et al. 2024). Although most implementation research originates from the United Kingdom and the United States, broader international studies are needed to understand implementation processes across diverse healthcare contexts (Villarosa et al. 2019). To address this gap, the present study focussed on the context of Dutch dentistry. Developing effective implementation strategies requires systematically mapping current care practices and identifying potential barriers and facilitators experienced by OHCPs when treating children and parents (Braspenning et al. 2015; Broadbent et al. 2008; Damschroder et al. 2022). The results of this study may inform international efforts to strengthen guideline implementation and promote person-centred, evidence-based care.

Objectives of this study were to (1) describe the current use of preventive and minimally invasive caries treatment; (2) evaluate their alignment with the national guidelines; and (3) identify barriers and facilitators experienced by OHCPs and parents in managing caries, in Dutch children under 12 years.

## Methods

This was a qualitative study using individual semi-structured interviews with parents of children under 12 years of age, prevention assistants, dental hygienists, and dentists between March 2024 and February 2025. The medical ethical review committee of the Radboudumc reviewed the study protocol and judged that due to its limited invasive nature, it was exempt from medical ethical review (CMO Arnhem-Nijmegen 2023–16982). The study was performed in accordance with the principles of the Declaration of Helsinki. Informed consent was obtained from participants before the interview. Results were reported as per the consolidated criteria for reporting qualitative research (COREQ) (Tong et al. 2007).

### Participants and recruitment

#### Dental practices

Dental practices in the Netherlands were purposively recruited via the Dutch Dental Hygienists Association through posts in their newsletter and social media. In addition, OHCPs who had participated in previous research and indicated they were open to be approached for future research were contacted directly (Elsenberg et al. 2025). Practice-level inclusion criteria were a patient population with ≥ 100 children (< 12 years) and employment of dentists, dental hygienists, and prevention assistants. Geographic diversity and variation in type of practice were pursued, recruiting practices affiliated with a dental chain organisation, youth dental care organisation, and unaffiliated general practices.

#### Oral healthcare professionals

Practice managers of participating practices were requested to nominate two OHCPs and one parent for participation in the interview study. The researcher subsequently received the contact details of the nominated participants. All dentists, dental hygienists, and prevention assistants working in the included dental practices were eligible to participate in the study, and initially, those nominated by their practice were included. Subsequently, additional OHCPs were purposively recruited through the participating practices considering diversity in occupation, work experience, and age. All OHCPs who were contacted agreed to participate. Data collection continued until sufficient variation in participating OHCPs was reached for each target group (dentists, dental hygienists, and prevention assistants) separately, and saturation was reached. Data saturation was assumed to be reached when little or no new subthemes were found in the data, which typically occurs after between 9 and 17 interviews (Francis et al. 2010; Hennink et al. 2022).

#### Parents

Parents with children under the age of 12 were recruited through the included dental practices. Study information with contact information of the researcher was displayed in the waiting room and OHCPs actively solicited parents to participate in the study. In selecting parents, variation was pursued in socioeconomic status, single- and two-parent families, and cultural background. All parents who were contacted agreed to participate. Saturation was assumed to be achieved when the defined variation in parents was included, and no new subthemes were determined by coding (Francis et al 2010).

### Semi-structured interviews

Interviews were conducted through videoconferencing using Microsoft Teams at a time convenient to the participant by a PhD student (MSc, dental hygienist), female, trained in qualitative research, and interviewing skills. The interview duration was approximately 45 min for OHCPs and 30 min for parents. Semi-structured interview guides were developed for OHCPs and parents separately, using a thematic structure based on the national guideline 'Oral care for children; prevention and treatment of caries' and Flottorp's implementation schedule (Flottorp et al. 2013) (including health system questions). To optimise the quality and relevance of the interview guide, it was reviewed by two researchers with extensive knowledge of qualitative research, and pilot interviews with one OHCP and one parent were conducted, of which results are not included in the study.

In the interviews with OHCPs, the following topics were explored: (1) demographic characteristics and professional background, (2) organisation of oral healthcare, (3) caries management, (4) person-centred care, (5) general barriers, (6) general facilitators, (7) familiarity and experience with the national guideline (Kennisinstituut 2020), and (8) barriers and facilitators to using the guideline. Interviews with parents included questions about: (1) demographic characteristics of parents and children, (2) experiences in oral healthcare practice, (3) caries management, (4) person-centred care, (5) barriers, and (6) facilitators for oral home care.

### Qualitative data analysis

Interviews were recorded, transcribed verbatim, and anonymized. Interviews were coded through thematic deductive and inductive (emergent themes) analysis in ATLAS.ti version 23. Flottorp's implementation scheme was used as the basic coding framework (Flottorp et al. 2013), with new codes and themes added where relevant. Two researchers (CE and SJ) independently generated the initial codes for each interview. Subsequently, codes were discussed to achieve consensus and calibration. A third researcher (LK) was consulted to resolve any discrepancies. After 7 OHCP and 5 parent interviews, the remaining interviews were coded by either CE or SJ and reviewed by the other coder. Any discrepancies were discussed with, if necessary, a third researcher (LK) involved. Initial codes were merged into thematic code groups and summarised in subthemes. This iterative process of analysing data and refining thematic codes and defining themes was thoroughly examined and rigorously discussed by CE, SJ, and LK. The final codebook and theme overview was presented to the research team including the independent researchers (CE, SJ, and LK), a professor of cariology and endodontology (MH), and a professor of paediatric dentistry and preventive dentistry (MV) and discussed to refine and finalise results.

## Results

Results are presented first for OHCPs and later for parents.

### OHCPs

Of 26 OHCPs included, 10 (38.5%) were dentists, 7 (26.9%) dental hygienists, and 9 (34.6%) prevention assistants. Most were under 40 years of age (80.8%) and female (88.5%). The majority graduated less than 15 years ago: 34.5% graduated after 2020 and 42.6% between 2010 and 2020. There was geographic diversity in large, middle, and small towns and variation in type of practice; see Table 1.

**Table 1:** Oral healthcare professionals characteristics, N = 26

Occupation, N (%)		
Dentist	10	(38.5)
Dental hygienist	7	(26.9)
Prevention assistant	9	(34.6)
Sex, N (%)		
Female	23	(88.5)
Male	3	(11.5)
Age (years), N (%)		
< 30	13	(50.0)
30–40	8	(30.8)
40–50	1	(3.8)
> 50	4	(15.4)
Year of graduation, N (%)		
> 2021	9	(34.6)
2010–2020	11	(42.3)
2000–2010	1	(3.8)
< 1999	5	(19.2)
Type of practice(s), N (%)		
General oral health practice	7	(26.9)
Dental practice chain	11	(42.3)
Youth dental care	8	(30.8)
Location Netherlands, N (%)		
Large city (> 100.000 inhabitants)	11	(42.3)
Middle large city (50.00–100.00 inhabitants)	5	(19.2)
Small town (< 50.000 inhabitants)	10	(38.5)

**Fig. 1:**
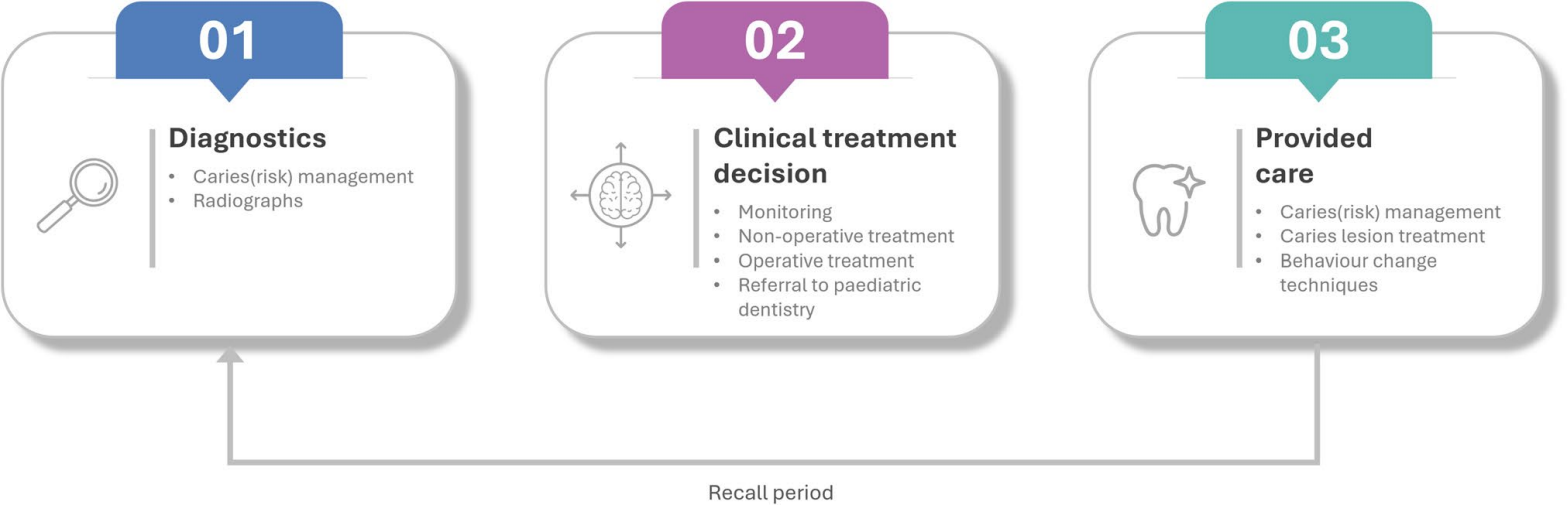
Caries management

The interviews explored the current oral care for children regarding caries (risk) management and caries management techniques, including diagnostics, clinical treatment decisions, and the provided care; see Fig. 1.

#### Diagnostics

**Figure:**
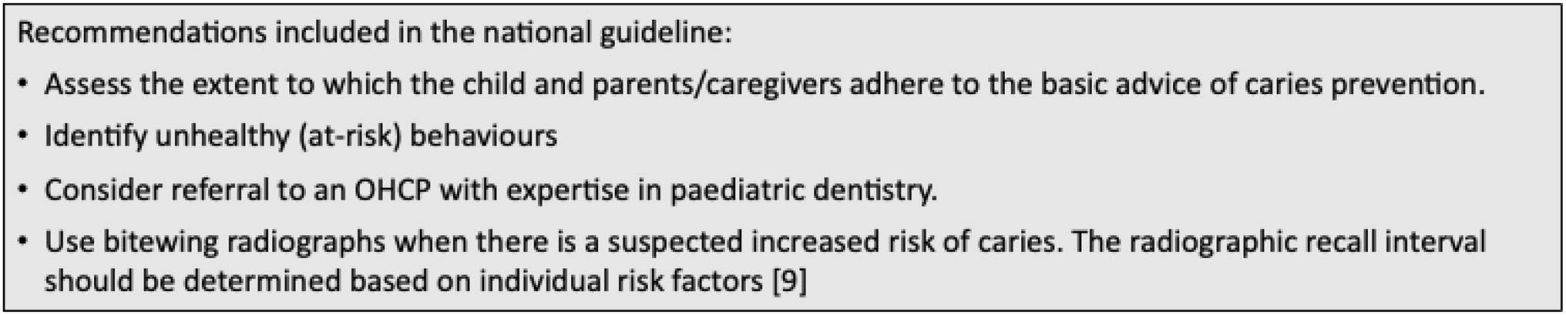

Adherence to brushing and eating advice was assessed at every visit, only during intake or only when caries was diagnosed. Caries risk factors were identified through history taking, sometimes using the ‘Gewoon Gaaf’ method. Radiograph use and frequency depended on factors like eruption of permanent teeth, caries history, caries activity, caries risk, interdental spaces and family caries. Some took a few radiographs, whilst others did so routinely at intake, at specific ages (3–7 years), or with first permanent molar eruption. Intervals ranged from 6 to 18 months depending on caries activity, or 2 to 3 years by default without caries activity. “*If everything looks good otherwise, the recall interval can be extended to two years.*” (Interview #17, dental hygienist, age 41).

#### Clinical treatment decision

Treatment decisions were said to depend on factors, such as the child’s age (eruption of permanent teeth), level of cooperation, dental anxiety, oral hygiene, fluoride use, dietary habits, child and parental motivation, parental preferences, aesthetics, cultural and medical factors, home environment, and the severity, location, and stadium of caries lesions. Also, perceived effectiveness of caries management and OHCPs’ personal preferences play a role. Some noted that shared decision-making is used when discussing treatment options. Children attending without parents may receive more invasive treatments due to reduced preventive effectiveness.

When initial lesions are diagnosed, some OHCPs emphasised arresting progression through self-care and professional interventions like fluoride application. Others applied invasive caries treatment options: *“If it's just enamel-dentine junction, so it's a small lesion, so really just through to the dentin, then often I choose to put a filling”.* (Interview #4, dental hygienist, age 25).

All OHCPs refer to specialised paediatric dentistry, with frequency and timing depending on local availability. Some OHCPs referred most children with caries lesions, whilst others referred only in cases of limited cooperation, anxiety, young age, trauma, disability, behavioural disorders, lack of time for acclimatisation, or to emphasise the seriousness of caries risk to parents. Referrals also occurred at parental request, in cases of high rate of caries lesions, or when general anaesthesia was needed.

#### Recall interval

National guidelines recommend individualised recall intervals based on caries risk assessment and set with parents, but a standard 6-month routine check-up was often used. *“If no additional findings are noted, the recall interval is six months”* (Interview #17, dental hygienist, age 41). Children with caries activity or higher risk were frequently scheduled for additional check-ups, becoming accustomed to the dental setting and/or fluoride application. Some extend the interval if oral health improves or the child adapted well. A caries risk score was sometimes used, but others have no system to set a recall interval or decide in consultation with parents.

#### Provided care

**Figure:**
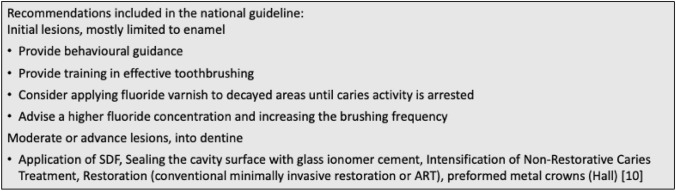

Some OCHPs deviate from the guideline by not educating parents on fluoride in toothpaste or routinely applying fluoride varnish on non-affected primary teeth. When caries lesions were detected, OHCPs reinforced preventive advice and gave additional advice to families with poor oral hygiene or caries activity, sometimes using plaque disclosing agents or photographs to visualise the dental plaque and applying fluoride varnish. They focus on improving parental motivation, promote self-care, and monitor compliance, repeating instructions if necessary. Several non-guideline-compliant practices were reported, such as sealing primary molars or using slicing (with or without Silver Diamine Fluoride (SDF)) for initial lesions.* “SDF application after slicing. And then you show to parents, now you can keep the surface clean properly. This treatment is sufficient, and then we never have to intervene anymore, and things don’t get complicated”* (Interview #10, dentist, age 65). Certain care, such as plaque disclosing application and standard twice-yearly fluoride application, was performed by default rather than based on individual indication.

OHCPs primarily use invasive treatments for caries lesions into dentine: primary dentitions are usually restoratively treated with composite, or alternatively with glass ionomer cement, for advanced lesion or limited child cooperation. However, some OHCPs never use glass ionomer cement: *“No, actually never [use glass ionomer]. I actually don't quite see more function, added value of that. What was that again?”* (Interview #20, dentist, age 31). Preformed metal crowns were used in cases of poor cooperation, limited motivation, extensive or advanced caries lesions, though opinions varied. Some reported negative outcomes (e.g., extraction), whilst others noted benefits, such as protecting permanent molars, non-retentive contacts, and avoiding anaesthesia. Slicing was also commonly applied, seen as effective for behaviour change and used by some for moderate caries: *“If it's still a non-cavitated lesion, then we'll indicate that brushing is very important and again cut down on those lemonades and all the sweets and stuff. Then we're just going to keep an eye on it. If it's approximal, and I see it shadowing due to underlying caries, then I will decide more quickly to slice”* (Interview #10, dentist, age 65). Some OHCPs were familiar with slicing but chose not to use it in children: *“So I don’t do slicing. I know that it is a good treatment option, but somehow, I don’t have that option in my workflow”* (Interview #20, dentist, age 31). The same applied to preformed metal crowns. Moderate caries lesions were sometimes monitored, especially with motivated parents. *“If it’s really just a tiny early-stage cavity, I sometimes just keep an eye on it, especially if I see that the oral hygiene is good, and the parents are cooperating”* (Interview #19, dentist, age 27).

#### Behaviour modification techniques

Showing parents problems such as dental plaque or caries lesions and providing education on brushing and nutrition was often reported and considered as behaviour change interventions. Most OHCPs did not use structured behaviour change methods. “*No, I don’t use a specific method. I mainly just talk and provide information*” (Interview# 21, prevention assistant, age 32). Encouraging self-care was considered challenging in young children, low SES families, parents with multiple children, or language barriers. A few OHCPs used motivational interviewing or the ’Gewoon Gaaf’ method, emphasising that the responsibility lies with the parents, or let parents come up with their own ideas and solutions. Some OHCPs adopted a collaborative approach, seeking compromises between their professional preferences and the family needs, showing understanding and interest, giving compliments and asking open-ended or follow-up questions. They avoided standardised narrative and focussed on small steps.

#### Practice organisation

Knowledge of local protocols was limited. The ‘Gewoon Gaaf’ system was implemented in several practices and believed to be more frequently used by younger OHCPs. Its adoption was reported to lead to improved teamwork, clarity, increased preventive care, and changes in recall periods. Dentists are responsible for caries management, though tasks are sometimes delegated. Prevention assistants mainly see children for preventive care and familiarisation with dental visits. They typically handle behaviour change-focussed appointments, as they are considered more skilled and have more time. Preventive and invasive treatments are often scheduled in parallel within one practice. Optimal oral hygiene was required before starting restorative treatments. *“Placing the responsibility on the parents, I will give them an appointment in a few weeks to see how the brushing is going. If it is going well, I will schedule an appointment to treat the molars.”* (Interview #1, dental hygienist, age 66). Deviations from protocols occurred due to OHCPs’ shortages, language barriers, behavioural disorders, uncooperative children, low parental motivation, absent parents, or ill-fitting preformed metal crowns.

#### Training

OHCPs reported developing communication and behaviour modification skills mainly through work and life experience. Some received training in communication, behaviour change techniques, and shared decision-making during formal or in continuing education (e.g., “Gewoon Gaaf”). A few were trained in the Hall technique and SDF. Continuing education on behaviour modification techniques was rare and often outdated, except in youth dental care where annual training was encouraged by management. Most OHCPs felt insufficiently competent in behaviour modification techniques and wanted more training, particularly in motivational interviewing. Challenges to applying this effectively included shifting responsibility to parents, the time required and frustration with repeating the same information. One OHCP believed that not all professionals are suited for these techniques, whilst another stressed their importance for long-term caries prevention in permanent teeth. *“When a child about five or six years old visits the dental practice, and there are lesions all over the deciduous teeth, then I am not going to fill everything. Then there must be a change first, otherwise al the permanent molars will soon be broken as well.”* (Interview #2, dentist, age 38).

#### Barriers

**Fig. 2:**
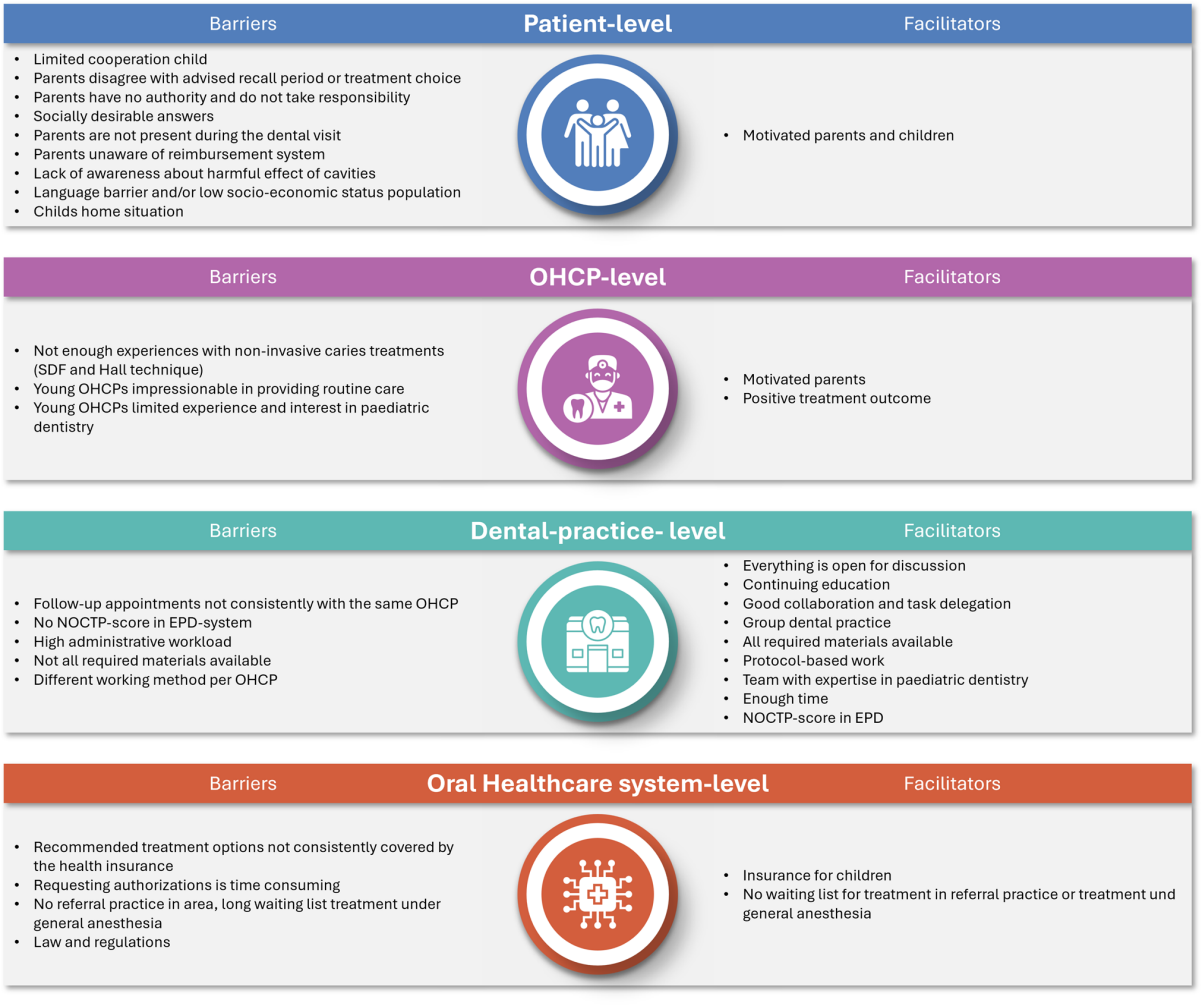
Barriers and facilitators to providing oral care for children: OHCPs’ perspectives

Barriers to providing dental care to children are visualised in Fig. 2. The only child-related barrier was limited cooperation, whilst parent-related barriers included lack of authority, responsibility, or awareness of the harms of caries, disagreement with recall intervals or treatment plans, and unfamiliarity with reimbursement.

Lack of experience with non-invasive caries treatments was reported and older OHCPs were considered less prevention-focussed. *“For those over 60, bringing prevention to the forefront for them doesn’t happen […] It’s a lost cause”*. (Interview# 20, dentist, age 31). Younger OHCPs were seen as more susceptible to providing standard care and less experienced or interested in paediatric dentistry. Several OHCPs noted a general operational mindset amongst colleagues, with a limited focus on behaviour change. “*They're all very operational and hands-on. We're all do-ers, so we just want to get things done and take action. We're not really in the habit of asking children questions like, what would you do, what could you come up with yourself? Or how important do you think your teeth are.”* (Interview# 23, dental hygienist, age 60).

Practice-level barriers included limited OHCPs continuity, no electronic patient record (EPR) integration of caries risk-score, high administrative workload, lack of necessary materials, and inconsistent practices amongst OHCPs. Workforce shortages were also noted: “*Because up there in the north [-ern region of the Netherlands], what about finding suitable oral health professionals? Now, that's very difficult”.* (Interview #9, prevention assistant, age 41).

Oral healthcare system-level barriers included lack of reimbursement for guideline-recommended treatments, restrictive laws and regulations, and misaligned financial incentives*. “You better do very intensive dental treatments in dentistry, because that's where you get rich.”* (Interview #10, dentist, age 65). Additionally, some regions lacked referral practices or had long waiting lists for general anaesthesia.

#### Facilitators

Motivated parents and children were identified as patient-level and OHCPs-level facilitators. OHCPs facilitators also included encouragement from positive patient outcomes. At practice level, facilitators included open communication, continuing education, collaboration, task delegation, protocol-based work, EPR risk score integration, time, and materials. At system level, insurance coverage and no waiting lists for referral practices or general anaesthesia were noted. “*What definitely helps is that parents don’t have to pay anything, oral healthcare is free up to the age of 18. I think that if that weren’t the case, fewer people would show up and people would be less willing to cooperate.*” (Interview #25, dental hygienist, age 33).

#### Barriers and facilitators for the national guideline

Barriers to applying the national guideline included its length, lack of specificity, time and administrative burden, and limited reimbursement. Many OHCPs were accustomed to working based on their own expertise rather than adhering to a guideline, noting that adhering to the guideline did not effectively halt caries progression. *“I found it and still find it very difficult, the national guideline. Whereas we used to treat much earlier, we are now indeed somewhat reticent or at least work with it. But I can also see that we really missed the mark in the beginning.”* (Interview #5, prevention assistant, age 56).

Facilitators included the guideline support for preventive and less invasive care, earlier recalls, structured support, and collaboration. Familiarity with the guideline varied, with few reporting thorough knowledge.

### Parents

A total of ten parents were included from various regions of the Netherlands and types of dental practices, representing a range of family compositions (including single-parent households and families with between one and four children), children’s age (range 0–12 years), and included children with sensory processing sensitivity, dental anxiety, and clinical conditions, such as Hypomineralised Second Primary Molar (HSPM).

Children saw various OHCPs, and whilst parents were generally satisfied, they noted unclear provider roles and different affinities for working with children. Parents trusted task delegation and professional scope and competencies. “*I assume she is qualified to do it, otherwise, I assume it would not be permitted.*” (Interview #2, mother of one child, age 43). They experienced individualised care based on the child’s clinical conditions, age, and personal circumstances, and reported preparatory sessions to familiarise children with dental treatment.

Most parents knew and generally followed basic caries prevention advice, such as twice-daily brushing and parental brushing, though some skipped brushing to avoid conflict. Guidance on diet and the timing of the first dental visit was less common, and children received varied professional dental care across the full spectrum.

**Fig. 3:**
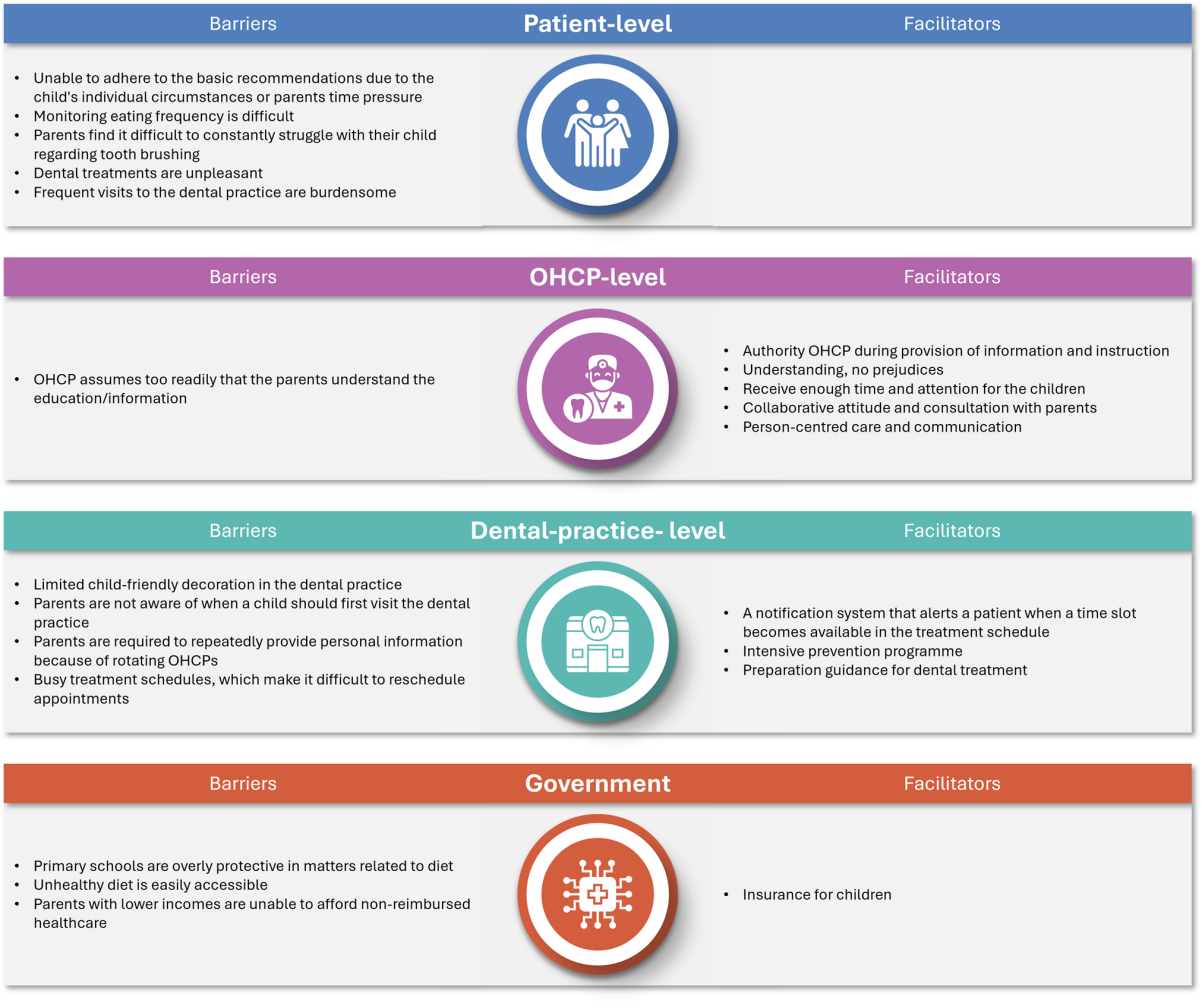
Barriers and facilitators for oral home care in children: the parents’ perspectives

Treatment options were inconsistently discussed, though parents were informed when radiographs were taken. Fluoride use varied by clinical indication (e.g., HSPM or caries activity), but was sometimes applied as part of routine care, with frequency ranging from every 6 weeks to once a year. Plaque disclosing agents were commonly used, often combined with professional cleaning. Recall intervals ranged from 6 weeks to 6 months, depending on individual factors. Some parents reported receiving no additional advice even after a caries diagnosis. Despite awareness of oral hygiene, parental brushing, and diet, behaviour change was not always achieved. Most parents felt responsible for their child’s oral health, and gradually shared this responsibility as children aged (> 10 years). “*You don’t visit the dentist without reason, so the dentist also plays a small role. However, they cannot control when you brush your teeth, that is your own responsibility. The same applies to limiting sugary snacks.*” (Interview #2, mother of one child, age 43). They expected adequate guidance from OHCPs when oral health declined (Fig. 3).

#### Barriers and facilitators from the parents’ perspective

At the patient level, basic advice was often not followed due to the child's individual needs or lack of time. Many parents found daily brushing a struggle and reported difficulty monitoring eating frequency, partly due to the easy availability of unhealthy food. *“All you can do is prepare them and explain that they should eat sweets in moderation. But you know, they have pocket money, so then I see another bag of candy lying around again*.” (Interview #10, mother of two children, age 47) 

Conversely, some parents felt that primary schools were overly protective about children’s diets. “*I think they’re going overboard in primary school now, not letting kids bring treats anymore. It always has to be healthy snacks—I don’t really get it.”* (Interview #10, mother of two children, age 47). Parents reported that OHCPs too readily assumed that they understand the education or information given during the dental check-up. “*What I notice at the dentist is that they sometimes just assume you understand it or know how to do it yourself.”* (Interview #3, mother of two children, age 38). Frequent dental visits were seen as burdensome, and treatments as unpleasant. Dental practice was not considered child friendly, and it was difficult to schedule sessions due to limited short-notice availability. Parents found repeatedly providing personal information due to rotating OHCPs unpleasant. They were also unaware of when their child should first visit the dentist and noted that lower-income families struggle to afford non-reimbursed care.

Facilitators included OHCPs’ professional, empathetic and non-judgmental approach, person-centred communication, and collaboration on treatment options, and for some, a directive style in delivering information and instructions. *“They could actually practice flossing with the kids sometimes, instead of me having to teach them. Because, having that authority is definitely an advantage.”* (Interview #3, mother of two children, age 38). The intensive preventive treatment programmes, treatment preparation, and children’s health insurance coverage were facilitators.

## Discussion

This study aimed to describe the current use of preventive and minimally invasive caries treatment for children in oral care practice in the Netherlands and to evaluate the extent to which these practices align with the national guidelines. Although OHCPs do address oral hygiene and eating habits, as recommended, their approach tends to emphasise the delivery of information rather than actively supporting behavioural change in parents and children. In general healthcare, it is well established that providing traditional advice is less effective compared to the use of behaviour change techniques, such as Motivational Interviewing (MI). MI has been shown to be effective in the previous studies, not only in initiating behaviour change but also in maintaining health-related behaviours, including those related to the prevention and management of oral diseases (Curtin et al. 2013). Most OHCPs did not use structured behaviour change methods, possibly due to the fact that they feel insufficiently competent in applying them. Many had not received recent training and there was a strong expressed need for further education, especially in MI. Full adoption of the national guideline, including MI, is required and targeted dissemination and implementation could be support achieving this (Curtin et al. 2013).

Many OHCPs have limited time to discuss health behaviours with patients, often prioritising short-term clinical goals over opportunities to support lasting behaviour change (Curtin et al. 2013). However, long-term behaviour change is essential for achieving and maintaining optimal oral health. Effective healthcare must centre on the individual and consider social and environmental factors beyond the dental office. Since medical care, genetics, and biology account for less than one-third of health determinants, addressing individual behavioural and lifestyle factors is critical (Lee et al. 2018). The national guideline promotes a patient-centred care model that respects and responds to individual needs and values, guiding clinical decisions accordingly. This approach has been linked to lower healthcare costs, better patient outcomes, and more effective behaviour change (Lee et al. 2018). In dentistry, integrating person-centred care is vital for preventing and managing caries (Kennisinstituut 2020) and it holds substantial promise for improving the quality of care and overall health outcomes (Lee et al. 2018).

Dental care has predominantly relied on invasive treatment-focussed approaches, with limited emphasis on disease prevention. However, emerging care models are reshaping the dental care system, shifting towards a system centred on disease management and prevention-oriented primary care that acknowledges patients' overall health and well-being (Lee et al. 2018). In line with this shift, the national guideline recommends that for initial or moderate lesions in primary dentition, the advice is to intervene as minimally invasively as possible. However, results of our study show that invasive interventions, such as a traditional composite restoration, are still commonly used. The translation of evidence into routine dental practice has been shown to be a gradual and complex process according to the previous research (Sbaraini et al. 2013). OHCPs are required to adjust their clinical routines to align more closely with the evidence-based recommendations (Sbaraini et al. 2013). Minimally invasive approaches, such as slicing or the application of preformed metal crowns (Hall technique), have been demonstrated to be equally effective in managing caries in the primary dentition (Schwendicke et al. 2021; Zaffarano et al. 2022). A possible explanation for this discrepancy is that many included OHCPs have limited experience with minimally invasive treatment strategies or lack the necessary materials, which reduces their willingness or ability to implement these approaches. Improvements are needed, and targeted educational implementation may help achieve this.

Several additional barriers and facilitators were reported by OHCPs and parents in managing caries in children under 12, including limited cooperation from the child, parental factors, a shortage of OHCPs, and the reimbursement system. A key barrier is that OHCPs report parents often fail to take responsibility for their child’s oral health and frequently disagree with the recommended recall interval or treatment plan. Furthermore, they perceive parents as being unaware of the potential risks their behaviour poses for the development of caries. Conversely, parents report that OHCPs tend to assume too readily that they understand the advice given, whilst many parents struggle to follow the basic advice about brushing and dietary habits. OHCPs tend to underuse behaviour change strategies, despite their proven effectiveness in improving treatment adherence. One possible explanation is that they have received inadequate training in behaviour change strategies. As a result, participants may lack the necessary support to adequately assist parents in need. Whilst full adherence to oral health advice is rare (Umaki et al. 2012), many parents still see themselves as responsible for their child’s oral health. The oral health care of children should be viewed by both parents and OHCPs as a collaborative process, in which each party holds specific responsibilities.

The findings of this study have several important implications for both future research and clinical practice. Implementing a new way of working does not usually occur automatically. Previous implementation research has shown that OHCPs are unlikely to change their clinical practices based solely on the availability of convincing evidence. To achieve a meaningful shift towards more preventive care, leadership within the dental practice is essential, along with a focus on cultural, social, and economic resources that shape the practice environment (Sbaraini 2012). Targeted implementation strategies that address the identified barriers may be required. Based on the results of our study, such strategies should focus on changing the behaviour of the OHCPs. Therefore, the strategies targeted on (1) increasing knowledge about the content of the guideline, (2) motivating by adjusting the attitude of OHCPs about responsibility, and (3) providing training in minimally invasive treatments, behaviour change techniques, and person-centred care. Research has suggested that behaviour change in primary care providers can be effectively supported through interactive, small-group educational formats (Close et al. 2010). Additionally, the result suggests that parents should also be a target group for these strategies. Strategies should inform them about the reimbursement system, what the basic recommendations are to prevent caries, what the harmful effects of caries are (including in the primary teeth), the recommended age for a first dental visit, and the importance of attending appointments with their child. Implementation could potentially occur at well-baby clinics, where it had been shown effective in the Netherlands (Van Spreuwel et al. 2025).

Although this qualitative interview study provided valuable insights into the experiences of OHCPs and parents, there are also some limitations. There may be inclusion bias, as practices were recruited through an open call, reducing their applicability to larger or more diverse populations. Additionally, a relatively high proportion of the participating practices were affiliated with dental chains. However, as this is a growing trend, it may well reflect future circumstances. Furthermore, the sample included OHCPs with a relatively low average age, which may have influenced their perspectives and approaches.

## Conclusion

Whilst OCHPs aim to apply preventive and minimally invasive caries management, alignment of their practices with the national guideline is limited, and several barriers are experienced. These findings offer actionable recommendations to improve paediatric oral health practice through better guideline implementation.

## Data Availability

The data that support the findings of this study are not publicly available due to their containing information that could compromise the privacy of research participants but are available from the corresponding author upon reasonable request.
